# Rectal Feeding after Abdominal Operations

**Published:** 1902-01

**Authors:** 


RECTAL FEEDING AFTER ABDOMINAL OPERATIONS.

HERRICK, J. B. (*Chicago Medical Recorder*) and WATSON, L.H. (*Medical Review of Reviews*) hold that much of the success of abdominal surgery at the present day is attributable to the great care bestowed in the preparation of the patient for operation and the after-treatment. As in many instances the nutrition is more or less seriously impaired, the question of alimentation assumes great importance in the after-management of the case. After operations upon the stomach and intestinal tract it may be, and often is, hazardous to feed the patient by mouth for some time and rectal feeding becomes indispensable. In selecting foods for nutritive enemas the point to be borne in mind is that the mucous membrane of the lower portion of the intestinal canal has but little digestive power, and hence the nourishment must be presented in such form that it can be easily absorbed. Another point is that the mucous membrane of the lower bowel soon becomes irritable unless the nutritive material is perfectly bland and also in such condensed state as to leave behind no residue to decompose and act as an irritant. Physiological experiments have shown that when albuminous material is transformed into albumoses it is absorbed almost immediately without requiring any preliminary digestion, and after being taken up into the circulation is rapidly reconverted into serum albumin. For this reason the albumoses are well adapted for rectal feeding, and according to the observations of Dr. J. B. Herrick, of Chicago and of Dr. L. H. Watson, of Chicago, somatose, which is a pure preparation of albumoses, is an excellent nutrient for this purpose. It may be administered alone in solution, or in connection with other foods, such as milk and white of egg.

In the article on Rectal alimentation, referred to, Dr. Watson recommends that the bowels should be emptied by a preliminary laxative or cleansing injection before administering the nutritive enema. The enema should be given at regular intervals through a soft rubber tube, and should be inserted rather high into the rectum. The amount should not exceed four or five ounces. As soon as the gastric disturbances subside the patient may be given small quantities of food by mouth, gradually diminishing the number of rectal enemas.

